# 解析PD-1/PD-L1抑制剂分子结构差异与不良反应的相关性

**DOI:** 10.3779/j.issn.1009-3419.2020.102.17

**Published:** 2020-07-20

**Authors:** 同济 谢, 守正 王, 镨元 邢

**Affiliations:** 100021 北京，国家癌症中心/国家肿瘤临床医学研究中心/中国医学科学院北京协和医学院肿瘤医院 National Cancer Center/National Clinical Research Center for Cancer/Cancer Hospital, Chinese Academy of Medical Sciences and Peking Union Medical College, Beijing 100021, China

**Keywords:** 程序性死亡受体1, 程序性死亡配体1, 结构, 不良反应, Programmed cell death protein 1, Programmed cell death ligand 1, Structure, Adverse events

## Abstract

针对程序性死亡受体1（programmed cell death protein 1, PD-1）及程序性死亡配体1（programmed cell death ligand 1, PD-L1）的免疫治疗作为一种新兴的方法在恶性肿瘤的治疗中起到越来越大的作用，相较于传统的化学治疗体现出更好的疗效。然而在应用针对PD-1/PD-L1的免疫检查点抑制剂的过程中也出现了许多不良反应，并且这些不良反应在不同药物中的发生率也不完全相同。由于区分不同药物的一个重要指标是它们的分子结构，故本文将从不同PD-1/PD-L1免疫检查点抑制剂的结构出发，通过综述不良反应的*meta*分析以及回顾性研究的结果解析分子结构与不良反应发生情况之间的相关性。

## Abstract (en)

As a new method, immunotherapy which is targeting programmed cell death protein 1 (PD-1) and programmed cell death ligand 1 (PD-L1) plays a more and more important role in the treatment of malignant tumors. Immunotherapy is more effective than traditional chemotherapy. However, there are also many adverse events during the application of immunocheckpoint inhibitors targeting PD-1/PD-L1, and the incidence rate of these adverse events among different drugs is different. Because the molecular structure of these drugs is an important indicator to distinguish them, this paper will analyze the correlation between molecular structure and adverse events of PD-1/PD-L1 immunocheckpoint inhibitors through reviewing some *meta*-analyses and retrospective analyses from the perspective of different structures.

程序性死亡受体1（programmed cell death protein 1, PD-1）/程序性死亡配体1（programmed cell death ligand 1, PD-L1）抑制剂是针对PD-1分子或是PD-L1分子的单克隆抗体，它们通过特异性地结合于各自的靶分子以阻断PD-1/PD-L1信号通路，从而达到解除肿瘤细胞对免疫细胞的负调节作用^[1]^。

目前针对PD-1分子的抗体大多属于免疫球蛋白G（immunoglobulin G, IgG）4亚类，例如Nivolumab、Pembrolizumab、Tislelizumab、Sintilimab、JS001、Camrelizumab等。在结构上，这些IgG4亚类的单克隆抗体除了具有各自的结构特点外，大多采用了将野生型IgG4亚类抗体的第228位丝氨酸替换为脯氨酸的改造^[2, 3]^，这一改造避免了IgG4亚类分子的“半分子交换”现象发生^[4, 5]^。“半分子交换”现象是指两个单特异性IgG4分子互相交换自身的一条重链以及与这条重链相连的轻链，从而产生新的IgG4分子的过程，而这种新的IgG4分子可能因此获得双特异性^[4, 5]^。这一现象与IgG4分子铰链区一个模体的氨基酸序列为C-P-S-C（半胱氨酸-脯氨酸-丝氨酸-半胱氨酸）有关，它赋予了IgG4分子更多的灵活性；在IgG1分子中，这一同源模体的序列为C-P-P-C（半胱氨酸-脯氨酸-脯氨酸-半胱氨酸），其脯氨酸限制了IgG1分子形成链内二硫键的能力，因此IgG1分子不存在“半分子交换”现象^[4]^。由于体内本身存在与抗肿瘤无关的IgG4分子（例如抗感染的IgG4分子），故检查点抑制剂与之发生“半分子交换”后会降低药物的亲和力^[3]^，而通过修改“C-P-S-C”为“C-P-P-C”可以避免交换的发生^[2, 3]^。

针对PD-L1分子的抗体大多属于IgG1亚类，例如Atezolizumab、Avelumab、Durvalumab等。在结构上，上述三种IgG1亚类的单克隆抗体除了具有各自的结构特点外，根据其可结晶片段（fragment crystallizable, Fc）是否保留IgG1亚类所具有的强抗体依赖的细胞介导的细胞毒性作用（antibody-dependent cell-mediated cytotoxicity, ADCC），可以进一步分为保留ADCC功能的Avelumab^[6]^与Durvalumab和去除ADCC功能的Atezolizumab^[7]^。

因此按照分子结构差异可以将上述目前临床上常用的5种药物大致分为3类：IgG4亚类单克隆抗体（Nivolumab, Pembrolizumab）、去除ADCC功能的IgG1亚类单克隆抗体（Atezolizumab）和保留ADCC功能的IgG1亚类单克隆抗体（Avelumab, Durvalumab）。本文将按照上述分类对这5种药物的分子结构特征进行描述，并汇总当前关于这5种药物不良反应发生情况的*meta*分析和回顾性研究结果，为药物设计及临床用药安全提供参考信息。

## 1 药物分子结构特征

在进行药物结构特征描述前，首先对药物作用的靶分子（PD-1/PD-L1分子）胞外段的主要结构进行阐述。PD-1分子胞外段的主要结构为从N末端至C末端依次排列的“A”、“B”、“C”、“C’”、“D”、“E”、“F”、“G”八个β折叠、连接它们的氨基酸所构成的各种“环”结构（如BC环、C’D环、FG环等）以及从N末端至“A”的结构（称为N环）^[8, 9]^；PD-L1分子胞外段的主要结构为从N末端至C末端依次排列的“A”、“B”、“C”、“C’”、“C'”、“D”、“E”、“F”、“G”九个β折叠、连接它们的氨基酸所构成的各种“环”结构（如BC环、CC’环、C’C"环、FG环等）以及从N末端至“A”的结构（称为N末端区域）^[10, 11]^。

### 1.1 IgG4亚类单克隆抗体

#### 1.1.1 Nivolumab

Nivolumab（纳武利尤单抗、纳武单抗）是Bristol-Myers Squibb公司研发的一种全人源抗PD-1分子的IgG4亚类单克隆抗体，于2014年12月22日被美国食品药品监督管理局（Food and Drug Administration, FDA）批准用于不可切除或转移性黑色素瘤的治疗，后又陆续获美国FDA批准用于肾细胞癌、非小细胞肺癌等的治疗。虽然单克隆抗体类药物的结构复杂，但仍可通过其与相应抗原即PD-1/PD-L1分子结合位点的不同来反映其抗原结合片段（fragment of antigen binding, Fab）段的结构差异。Tan等^[8]^的研究显示，Nivolumab的1个Fab段与PD-1分子胞外段之间共形成16个氢键，其中PD-1的“N环”结构贡献了10个氢键，是Nivolumab的主要结合位点，“FG环”结构提供了5个氢键形成位点，而“BC环”结构仅提供了1个氢键形成位点。该研究还进一步揭示了Nivolumab通过与“FG环”结构的结合以发挥干扰PD-1与PD-L1结合的作用，而Nivolumab与“N环”结构结合则为其发挥阻断功能提供了牢固的“停靠点”。

#### 1.1.2 Pembrolizumab

Pembrolizumab（帕博利珠单抗、派姆单抗）是Merck公司研发的一种人源化抗PD-1分子的IgG4亚类单克隆抗体，于2014年9月4日被美国FDA批准用于不可切除或转移性黑色素瘤的治疗，后又陆续获美国FDA批准用于非小细胞肺癌、头颈部鳞状细胞癌等的治疗。根据Na等^[9]^的研究，Pembrolizumab的Fab段与PD-1分子相互作用的接触面可以被分为两个部分，即两个亚界面。亚界面Ⅰ由PD-1分子的“C’D环”结构和Pembrolizumab互补决定区的L1、L2、H2以及骨架区的4个β折叠构成。PD-1分子的“C’D环”结构是Pembrolizumab结合的主要部位；亚界面Ⅱ由PD-1分子的“C”、“C’”、“F”的部分结构和Pembrolizumab互补决定区的L1、H3构成。PD-1分子的“C”、“C’”、“F”的部分结构是Pembrolizumab与PD-L1分子竞争结合的部位，即介导Pembrolizumab发挥阻断功能的部分。

### 1.2 去除ADCC功能的IgG1亚类单克隆抗体

Atezolizumab（阿替利珠单抗、阿特珠单抗）是Roche公司研发的一种全人源抗PD-L1分子的IgG1亚类单克隆抗体，于2016年5月18日被美国FDA批准用于尿路上皮癌的治疗，后又获FDA批准用于非小细胞肺癌、乳腺癌等的治疗。根据Lee等^[10]^的研究，Atezolizumab与PD-L1分子的结合主要依靠Atezolizumab的Fab段和PD-L1分子的“C”、“C’”、“F”、“G”所构成的“CC’FG反向平行β片层”结构，并且PD-L1分子的这一结构也是其与PD-1分子相互作用的关键结构，即Atezolizumab与PD-1分子竞争结合的部位。除此之外，PD-L1分子的“BC环”、“CC’环”、“C’C’’环”以及“FG环”结构也有助于Atezolizumab与其稳定地结合。

### 1.3 保留ADCC功能的IgG1亚类单克隆抗体

由Pfizer公司与Merck Serono公司联合研发的Avelumab（阿维鲁单抗、阿维单抗）以及由AstraZeneca公司研发的Durvalumab（度伐鲁单抗）均为全人源抗PD-L1分子的IgG1亚类单克隆抗体。Avelumab于2017年3月24日被美国FDA批准用于转移性默克尔细胞癌的治疗，后又获美国FDA批准用于尿路上皮癌的治疗。Durvalumab于2017年5月1日被美国FDA批准用于尿路上皮癌的治疗，后又获美国FDA批准用于非小细胞肺癌的治疗。根据Lee等^[10]^以及Liu等^[11]^的研究，Avelumab以及Durvalumab的Fab段亦主要结合于PD-L1分子的“CC’FG反向平行β片层”结构。除此之外，PD-L1分子的其他结构也有助于单克隆抗体与之结合：其中“CC’环”结构有助于Avelumab和Durvalumab与其稳定地结合；而“N末端区域”有助于Durvalumab与其稳定地结合。

## 2 不良反应

尽管按照结构进行分类可以将上述常用的5种药物分为三大类，临床研究中还是倾向于按照药物作用的靶分子的差异将其分为两类，即抗PD-1单克隆抗体与抗PD-L1单克隆抗体。当然这种常用的分类方式也可以理解为把这5种药物分为IgG4亚类组与IgG1亚类组。目前分析不同药物的不良反应发生情况的文献的结论可以归纳为两大类，即分析组内差异（主要是IgG4组的组内差异，即Nivolumab与Pembrolizumab之间的差异）与组间差异（IgG4组与IgG1组之间的差异）。

在不良反应差异的研究中最常见的两种亚组分析的因素是不良反应种类与肿瘤类型，在分析风险差异时应当综合考虑这两种因素，否则得到的结论将是粗糙和片面的。如Wang等^[12]^的*meta*分析中纳入了肺癌、胃癌、黑色素瘤等多种肿瘤以及皮肤、内分泌系统、呼吸系统等多个系统的不良反应，但并未按照肿瘤类型以及不良反应种类分析不同药物间的风险差异，只是得到不良反应的总体发生情况在PD-1组内无差异（全级不良反应：Nivolumab对Pembrolizumab的比值比为1.28，95%CI: 0.97-1.79）。再如Huang等^[13]^网状*meta*分析也未综合考虑这两种因素，得到治疗相关不良反应在PD-1组与PD-L1组之间无差异（全级治疗相关不良反应：PD-1组对PD-L1组的比值比为1.26，95%CI: 0.74-2.17）。当按照具体类型的不良反应进行分析后得到了比较详细的结论：不同药物间在肺炎（Nivolumab对Pembrolizumab的比值比为1.10，95%CI: 0.86-1.40）^[14]^、结肠炎（PD-1组发生率为2/9, 136，PD-L1组发生率为0/3, 164，计算得到*P*≈1）^[15]^的风险上无差异，在甲状腺功能亢进（Nivolumab发生率为2.5%，Pembrolizumab发生率为3.8%，*P*=0.04）^[16]^、皮疹（Nivolumab发生率为19.27%，Pembrolizumab发生率为7.66%，*P*=0.008）^[17]^的风险上存在差异，但Li等（*P*=0.21）^[18]^与Yang等（*P*=0.84）^[19]^的研究认为皮疹的发生风险在Nivolumab与Pembrolizumab间不存在差异，这提示不良反应的种类与不良反应的风险差异可能有关联；对于肿瘤类型这一因素，Duan等^[20]^的*meta*分析的研究对象包括非小细胞肺癌、肾细胞癌、尿路上皮癌等。其研究结果显示，胃癌患者接受单药治疗后所有种类不良反应的发生风险在PD-1组更高（PD-1对PD-L1的风险比为2.42，95%CI: 1.74-3.38），而尿路上皮癌患者则是PD-L1组风险更高（PD-1对PD-L1的风险比为0.87，95%CI: 0.77-0.98）。因此，肿瘤类型与不良反应的风险差异也可能有关联。

综上分析，不良反应的种类以及肿瘤类型与不良反应的发生风险均存在一定的关联，而单独分析其中一个因素是不全面的。为得到可靠的结论，本文进一步分析非小细胞肺癌患者在接受不同抗PD-1/PD-L1单克隆抗体治疗后不同类型不良反应的发生风险是否存在差异。

### 2.1 组内差异（表 1）

**1:** 非小细胞肺癌患者接受Nivolumab或Pembrolizumab治疗后不良反应风险差异 Differences of the risk of adverse events between Nivolumab group and Pembrolizumab group in non-small cell lung cancer patients

References	Hypothyroidism	Anemia	Pneumonitis	Leukopenia	Nausea	Arthralgia
[21]	OR^a^: 3.28 (0.06-4, 595.00)	OR^a^: 0.33 (0.13-0.78)	OR^a^: 4.28 (0.42-126.00)	OR^a^: 0.09 (0.00-0.84)	OR^a^: 0.56 (0.32-0.97)	OR^a^: 1.08 (0.46-2.44)
[22]	Incidence: 13.0% (Nivolumab) 4.9% (Pembrolizumab) *P* value wasn’t given.*	NA	Incidence: 4.8% (Nivolumab) 14.6% (Pembrolizumab) *P*=0.028	NA	NA	Incidence: 3.5% (Nivolumab) 12.2% (Pembrolizumab) *P*=0.032
[23]	NA	NA	OR^b^: 3.259 (1.361-7.807)	NA	NA	NA
[24]	NA	NA	RR^a^: 3.94 (0.65-23.79)	NA	NA	NA
[25]	OR^b^: 1.46 (0.06-33.70)	NA	OR^b^: 0.25 (0.03-1.74)	NA	NA	NA
The number in parentheses represent confidence interval; NA: this kind of adverse event was not analyzed in this article; OR^a^ : odds ratio of Nivolumab versus Pembrolizumab; OR^b^ : odds ratio of Pembrolizumab versus Nivolumab; RR^a^: risk ratio of Nivolumab versus Pembrolizumab. *The P value was not given in the article, but the data for calculating P value was listed. The *P* value was 0.189.						

组内差异的研究主要集中在IgG4亚类的两种药物的不良反应的风险差异。例如Almutairi等^[21]^的网状*meta*分析提示Nivolumab比Pembrolizumab具有更低的贫血、白细胞减低以及恶心的风险；Ksienski等^[22]^的多中心回顾性研究得出Nivolumab有更低的关节痛的风险，但是Almutairi等^[21]^的研究并未发现这种差异；Ksienski等^[22]^的研究提示Nivolumab的肺炎风险低于Pembrolizumab，Fukihara等^[23]^的回顾性研究进一步揭示Pembrolizumab的使用是肺炎的独立危险因素，但Almutairi等^[21]^、Passiglia等^[24]^及Peng等^[25]^的*meta*分析的结果提示肺炎的发生风险在Nivolumab与Pembrolizumab之间并无统计学差异（值得注意的是，尽管差异没有统计学意义，Passiglia等^[24]^的研究仍认为Pembrolizumab引起肺炎的风险低于Nivolumab有价值）。当然在另一些不良反应中Nivolumab与Pembrolizumab并未表现出有统计学意义的差异。如Almutairi等^[21]^以及Peng等^[25]^的研究均提示甲状腺功能减退的发生风险在两种药物之间无差异。

### 2.2 组间差异（表 2）

**2:** 非小细胞肺癌患者接受抗PD-1单克隆抗体或抗PD-L1单克隆抗体治疗后不良反应风险差异 Differences of the risk of adverse events between PD-1 group and PD-L1 group in non-small cell lung cancer patients

References	Colitis	Peripheral edema	Peripheral neuropathy	Pneumonitis	Neutropenia	Vomiting
[21]	OR^c^: 4.90 (0.04-37, 693.18) OR^d^: 0.78 (0.01-39.32)	OR^c^: 0.31 (0.12-0.72) OR^d^: 0.40 (0.17-0.88)	OR^c^: 0.26 (0.08-0.72) OR^d^: 0.30 (0.08-0.86)	OR^c^: 0.11 (0.00-2.18) OR^d^: 0.46 (0.00-26.36)	OR^c^: 0.18 (0.02-0.75) OR^d^: 0.10 (0.01-0.43)	OR^c^: 0.45 (0.23-0.88) OR^d^: 0.50 (0.25-0.98)
[26]^*^	Incidence (grade 3-5): 0.95% (PD-1), 0.35% (PD-L1), *P*=0.04	NA	NA	NA	NA	NA
[27]	NA	NA	NA	Incidence: 4% (PD-1), 2% (PD-L1), *P*=0.01	NA	NA
[28]	NA	NA	NA	Incidence: 3.6% (PD-1), 1.3% (PD-L1), *P*=0.001	NA	NA
[29]	NA	NA	NA	*P* value wasn’t given	NA	NA
[30]	NA	NA	NA	RR^b^: 0.28 (0.01-5.42) RR^c^: 1.05 (0.05-22.87)	NA	NA
The number in parentheses represent confidence interval; NA: this kind of adverse event was not analyzed in this article; OR^c^: odds ratio of Pembrolizumab versus Atezolizumab; OR^d^: odds ratio of Nivolumab versus Atezolizumab; RR^b^: risk ratio of Pembrolizumab versus Atezolizumab; RR^c^: risk ratio of Nivolumab versus Atezolizumab; PD-1: programmed cell death protein 1; PD-L1: programmed cell death ligand 1. ^*^Incidence of all grade colitis is 1.52% and 0.68% in PD-1 group and PD-L1 group respectively, *P*=0.06.						

接受IgG1亚类组单克隆抗体治疗的非小细胞肺癌患者与接受IgG4亚类组单克隆抗体治疗的患者间不良反应的发生风险也存在有统计学意义的差异。例如Lin等^[26]^的*meta*分析提示PD-L1组的3级-5级结肠炎的风险低于PD-1组，而在Almutairi等^[21]^的研究中并未发现这一差异；在Pillai等^[27]^以及Khunger等^[28]^的*meta*分析中发现PD-L1组的肺炎发生风险更低（Tartarone等^[29]^的*meta*分析也得出这一结论，但并未进行统计学检验），而Almutairi等^[21]^、Wu等^[30]^的*meta*分析却提示肺炎在两组中的风险差异不具有统计学意义；再如在Almutairi等^[21]^的研究中发现Atezolizumab的中性粒细胞减少、周围性水肿、呕吐以及周围神经病变的发生风险要高于Nivolumab和Pembrolizumab，即这些不良反应的风险在PD-1组更低。

## 3 分子结构与不良反应关系的解析与药物研发建议

综合以上对于IgG4亚类单克隆抗体之间不良反应发生风险差异的分析，可以得出Nivolumab具有更低的贫血^[21]^、白细胞减低^[21]^、恶心^[21]^、关节痛^[22]^以及肺炎^[22, 23]^风险的结论，这也许与两种药物的结构不同有关。评价药物与靶分子结合的亲和力的一个重要参数是解离常数（K_D_），单位为mol/L，其含义为A和B两种分子处于平衡状态时的解离程度，K_D_越大说明解离越多，A、B分子之间的亲和力越弱。Nivolumab与哺乳类动物的PD-1分子的K_D_为1.45 nmol/L^[8]^（1 nmol/L=10^-9^ mol/L），而这一数值大于Pembrolizumab的27 pmol/L^[9]^（1 pmol/L=10^-12^ mol/L），这说明Nivolumab与PD-1分子的亲和力更低。虽然没有研究证实，但可以推测由于两种药物结构不同造成了与PD-1分子的结合部位的不同，这种差异使得Nivolumab对PD-1分子具有相对较低的亲和力，而具有较低亲和力的Nivolumab将限制重新活化免疫细胞的数目，由于一些不良反应与过度重新活化的免疫细胞有关，因此Nivolumab的不良反应发生风险较低。但值得注意的是，仍有一些不良反应的风险如甲状腺功能减退在Nivolumab与Pembrolizumab之间并未发现差异^[21, 25]^，这提示单纯用结构的差异来解释不良反应的风险差异是不全面的。

关于组间差异，3级-5级结肠炎^[26]^、肺炎^[27, 28]^在PD-L1组的风险可能较小。在Duan等^[20]^以及Lin等^[26]^的研究中，这种现象被归因于抗PD-L1单克隆抗体只能阻碍PD-1与PD-L1分子的结合而不能干预PD-1分子与PD-L2分子结合。而抗PD-1单克隆抗体则可以阻断两条通路，故而不良反应风险高。当然，由于目前针对PD-1的抗体大多为IgG4亚类而针对PD-L1的抗体大多为IgG1亚类，故尽管没有研究验证，不良反应的风险差异或许与抗体的结构差异也存在一定关联；然而对于另外一些不良反应，如中性粒细胞减少、周围性水肿、呕吐以及周围神经病变^[21]^在PD-1组具有更低的风险，目前尚未找到合理的解释。

鉴于不同结构药物的不良反应风险存在差异，在药物研发上可以做如下探究：首先，目前针对PD-1分子的Nivolumab与Pembrolizumab显现出不同的PD-1分子亲和力^[8, 9]^与不良反应风险^[21-23]^，而不同的亲和力也可能造成药物疗效的差异（尚未得到证实），因此可以探究并设计适当结构的药物，使其与PD-1分子以较为合适的亲和力结合，从而在保证有效治疗效果的前提下尽量减小不良反应的风险；其次，由于Atezolizumab、Avelumab以及Durvalumab与PD-L1分子的作用位点有较多的重合^[10]^，可以研究各药物私有的结合部位对药物亲和力的影响，或者探讨ADCC作用的去留对不良反应风险的影响，从而设计低不良反应风险的PD-L1单克隆抗体；最后，可以进一步探究和研发IgG1亚类的抗PD-1抗体与IgG4亚类的抗PD-L1单克隆抗体，找到最佳的“靶点-抗体亚类”组合。

## 4 局限性

本文分析了关于PD-1/PD-L1免疫检查点抑制剂不良反应发生风险的17篇*meta*分析和2篇回顾性研究，但存在一些局限性：首先，这些分析和研究已经是对原始研究数据进行提取分析的文献，本文在此基础上进一步分析存在相关信息丢失的风险；其次，不同分析纳入的药物种类、患者数目、计算指标以及分析的方法等不尽相同，这使得可比性下降。例如在进行组间差异分析时，Almutairi等^[21]^的研究中PD-L1组仅纳入了Atezolizumab，而Tartarone等^[29]^的研究还纳入了Avelumab，Pillai等^[27]^的研究在纳入了Atezolizumab的基础上同时纳入了Avelumab与Durvalumab；最后，目前缺少对于IgG1亚类组内3种药物不良反应风险比对的研究，新的IgG4亚类单克隆抗体药物的试验数据也十分有限，这些使得进一步分析结构与不良反应风险之间的关系受到限制。

## 5 结论

根据对目前最常用的5种作用于PD-1/PD-L1通路的单克隆抗体药物的分析，可以得知不同药物具有不同的高风险不良反应，而这与它们的结构差异存在一定的关联。因此，在临床上使用上述5种药物以及其他作用于这一通路的药物时，从结构角度出发考虑其不良反应及安全性是一个值得选择的方向。
